# Financial Incentives for Healthy Living for Patients With Cardiac Disease From the Perspective of Health Care Professionals: Interview Study

**DOI:** 10.2196/27867

**Published:** 2021-08-30

**Authors:** David de Buisonjé, Jessica Van der Geer, Mike Keesman, Roos Van der Vaart, Thomas Reijnders, Jobke Wentzel, Hareld Kemps, Roderik Kraaijenhagen, Veronica Janssen, Andrea Evers

**Affiliations:** 1 Health, Medical and Neuropsychology Unit Leiden University Leiden Netherlands; 2 Department of Psychology, Health and Technology University of Twente Enschede Netherlands; 3 Department of Cardiology Máxima Medical Center Veldhoven Netherlands; 4 Hearts4people Foundation Amsterdam Netherlands; 5 Leiden University Medical Center Leiden University Leiden Netherlands; 6 Medical Delta Leiden-Delft-Erasmus Universities Delft Netherlands

**Keywords:** financial incentives, material rewards, healthy lifestyle, cardiovascular disease, cardiac rehabilitation, CVD

## Abstract

**Background:**

A promising new approach to support lifestyle changes in patients with cardiovascular disease (CVD) is the use of financial incentives. Although financial incentives have proven to be effective, their implementation remains controversial, and ethical objections have been raised. It is unknown whether health care professionals (HCPs) involved in CVD care find it acceptable to provide financial incentives to patients with CVD as support for lifestyle change.

**Objective:**

This study aims to investigate HCPs’ perspectives on using financial incentives to support healthy living for patients with CVD. More specifically, we aim to provide insight into attitudes toward using financial incentives as well as obstacles and facilitators of implementing financial incentives in current CVD care.

**Methods:**

A total of 16 semistructured, in-depth, face-to-face interviews were conducted with Dutch HCPs involved in supporting patients with CVD with lifestyle changes. The topics discussed were attitudes toward an incentive system, obstacles to using an incentive system, and possible solutions to facilitate the use of an incentive system.

**Results:**

HCPs perceived an incentive system for healthy living for patients with CVD as possibly effective and showed generally high acceptance. However, there were concerns related to focusing too much on the extrinsic aspects of lifestyle change, disengagement when rewards are insignificant, paternalization and threatening autonomy, and low digital literacy in the target group. According to HCPs, solutions to mitigate these concerns included emphasizing intrinsic aspects of healthy living while giving extrinsic rewards, integrating social aspects to increase engagement, supporting autonomy by allowing freedom of choice in rewards, and aiming for a target group that can work with the necessary technology.

**Conclusions:**

This study mapped perspectives of Dutch HCPs and showed that attitudes are predominantly positive, provided that contextual factors, design, and target groups are accurately considered. Concerns about digital literacy in the target group are novel findings that warrant further investigation. Follow-up research is needed to validate these insights among patients with CVD.

## Introduction

### Background

Despite the proven effectiveness of cardiac rehabilitation in initiating lifestyle change, many people with cardiovascular disease (CVD) fail to maintain a healthy lifestyle in the long term and relapse into unhealthy habits when they return to everyday life [1]. Therefore, there is an urgent need to find new approaches that increase the uptake of and long-term adherence to lifestyle interventions for patients with CVD [2]. A promising new approach is the use of financial incentives as a supplement to existing lifestyle interventions in CVD care. Financial incentives have not been applied often in the context of CVD care but have proven to be effective for a wide range of lifestyle behaviors that are relevant to CVD, including medication adherence, weight loss, smoking cessation, and physical activity [3-6]. However, implementing financial incentives for a healthy lifestyle remains controversial, and ethical objections have been raised [7]. For example, financial incentives can be perceived as paternalistic, coercive, involve bribery, or undermine the agency of the person [7]. Indeed, a recent systematic review on the acceptance of financial incentives for a healthy lifestyle has shown that acceptability is polarized and context dependent [8].

The acceptability of financial incentives has been studied often, among different populations, and with mixed results [8,9]. The most recently available systematic review on financial incentive acceptability concluded that “acceptability remains polarized, and [...] is shaped in complex and unpredictable ways” [8]. As an illustration of this polarization, incentives that specifically target deprived or vulnerable subgroups are found fair by some studies because they are a tool to redistribute resources to improve health among the disadvantaged [10]. In contrast, other research found a preference for generic incentives because targeted incentives were perceived as unfair to individuals who had already maintained a healthy lifestyle [11]. Although polarized, the available research has identified factors that consistently moderate the acceptability of financial incentives. Financial incentives appear to be more acceptable when they are privately funded, perceived as fair, (cost) effective, and when offered in the form of vouchers instead of cash [8,9,12,13].

Despite the variability found in acceptability, when we look specifically at the acceptability of financial incentives among health care professionals (HCPs), Hoskins et al [8] reported consistently high levels of acceptability. However, the authors point out that the reviewed studies were performed only in the United States, the United Kingdom, Australia, Canada, and France, which limits the generalizability of these findings. To the best of our knowledge, one study has specifically investigated the acceptability among HCPs working in CVD care in the United States [14]. This study showed that primary care physicians show a *broad and deep* acceptance of financial incentives. More importantly, this study showed that physicians’ perceptions of financial incentives were related to their patients’ clinical outcomes, thus emphasizing the importance of studying acceptability among HCPs involved in delivering the incentives.

To summarize, the acceptability of financial incentives is polarized, but reviews show indications of high acceptance among HCPs. At the same time, acceptability also appears highly dependent on the specific form and context in which financial incentives are offered and implemented.

### Objectives

To our knowledge, this study is the first to investigate the perspectives of Dutch HCPs on financial incentives as a supplement to CVD care in the Netherlands. We study the perspectives of HCPs because they are expected to deliver the intervention, promote its uptake among patients, and guide implementation in current health care in the Netherlands. This study addresses two research questions. First, what are HCPs’ attitudes toward using a financial incentive system for healthy living for patients with CVD? Second, what are the barriers and facilitators for implementing a financial incentive system as a supplement to existing CVD care?

## Methods

### Sample

A total of 16 semistructured, in-depth, face-to-face interviews were conducted between December 2017 and March 2018 with Dutch HCPs who support patients with CVD with living more healthily. In the Netherlands, the responsibility for supporting lifestyle changes in patients with CVD lies primarily with specialized nurse practitioners in hospitals, multidisciplinary professionals working in cardiac rehabilitation centers, and general practitioners and their assistants working in primary care. Therefore, we aimed to obtain diverse perspectives by including professionals with varying backgrounds from different institutions that are widely spread across the Netherlands (Table 1). After 16 interviews, no new information emerged, and data saturation was reached.

**Table 1:** Organization and professional background of respondents (N=16).

Organization and professional background		Respondents, n (%)
**Academic hospital A**		
	Nurse practitioner working in cardiac rehabilitation	2 (13)
**Academic hospital B**		
	Neurovascular nurse practitioner	1 (6)
	Physician assistant specialized in cardiovascular risk factor management	1 (6)
**Hospital A**		
	Physiotherapist working in cardiac rehabilitation	1 (6)
	Nurse practitioner working in cardiac rehabilitation	1 (6)
**Hospital B**		
	Physician-researcher working in cardiac rehabilitation	1 (6)
	Nurse practitioner working in cardiac rehabilitation	1 (6)
**Hospital C**		
	Neurologist specialized in cardiac rehabilitation	1 (6)
	Nurse practitioner working in cardiac rehabilitation	1 (6)
**Cardiac rehabilitation center A**		
	Cardiologist in residence	1 (6)
	Lifestyle coach working in cardiac rehabilitation	1 (6)
**Cardiac rehabilitation center B**		
	Physiotherapist working in cardiac rehabilitation	1 (6)
**Cardiac rehabilitation center C**		
	Psychologist specialized in cardiac rehabilitation	1 (6)
**General practice center A**		
	General practitioner specialized in cardiovascular disease care	1 (6)
	Nurse practitioner working in cardiac rehabilitation	1 (6)

### Procedure

We used convenience sampling and contacted individuals and organizations that were associated with the BENEFIT consortium. The BENEFIT consortium integrates care and noncare settings and connects public and private partners with the aim of scientifically evaluating the implementation of a reward system for healthy living for patients with CVD. Interviewees were contacted based on three criteria: (1) the interviewee had to be involved in lifestyle changes in patients with CVD, (2) we aimed to recruit HCPs from diverse professional backgrounds, and (3) we aimed to recruit HCPs from different organizations geographically spread across the Netherlands. We did not receive any explicit rejection to participate. Interview appointments were made by phone, after which the interviews were planned based on the location of the interviewee. Before the start of the interview, the interviewee was given a brief introduction about the project of which this interview was a part of, the goal of the interview, the procedure that would be followed and how the data would be processed. If the interviewee agreed and had no further questions, they signed an informed consent form, and the interview started. The interviews were conducted by 2 researchers (JVDG and DDB). One of the researchers led the interview, whereas the other was responsible for managing the audio recording and taking notes (these roles were alternated each time). The conversations were held in Dutch, audio recorded, fully transcribed, and finally pseudonymized to secure the privacy of the interviewees and possible relevant other people or organizations that were mentioned during the interviews. At the end of the interviews, the researcher summarized the key points covered and offered participants the chance to add to, revise, or clarify their views. Ethical approval for this study was obtained through a larger ethical approval process, which was required for the project at large. The main ethical concerns revolved around protecting the identity of individuals and the name of the organizations that were mentioned during the interviews, and we dealt with this by pseudonymizing the transcripts as described earlier.

A semistructured topic guide shaped the structure of the interviews, while allowing the interviewers to elaborate on the answers of the HCPs when relevant. This study reports on a subset of data related to the perceptions of HCPs on using a financial incentive system for lifestyle change in patients with CVD. This was one of the six themes discussed during the interviews. The other themes that were discussed, but not addressed in this study, were adherence of patients with CVD to a healthy lifestyle, supporting a healthy lifestyle, which stakeholders are involved in supporting a healthy lifestyle, using eHealth to support a healthy lifestyle, and using wearables and sensors to support a healthy lifestyle. Multimedia Appendix 1 contains the entire topic guide of the interviews. The interviews took an average of 60 minutes, of which approximately 15 minutes were spent on talking about an incentive system.

The interviewer first explained what the financial incentive system might look like. This explanation was based on the conceptual ideas developed within the BENEFIT consortium [15]:

With the BENEFIT program, patients can earn reward points for behaving healthily. For example, by going to their scheduled GP visits, but also by being physically active or self-monitoring their blood pressure. These reward points can then be exchanged for discounts on grocery items in the supermarket or to get a free healthy activity.

Then, the interviewer asked three questions: (1) What is your *opinion* on using an incentive system? (2) What could be *obstacles* for using an incentive system? and (3) What could be *solutions* to facilitate the use of an incentive system?

### Analysis

Transcripts were analyzed using a bottom-up inductive approach. This means that we made meaning out of the data itself instead of using a top-down theoretical approach with a framework or theory to which data were fitted. To structure the data analysis process, we followed the six steps of thematic analysis [16], which included (1) data familiarization, (2) generating initial codes, (3) searching for themes, (4) reviewing themes, (5) defining and naming themes, and (6) producing the report.

In each of the pseudonymized transcripts, 2 researchers (JVDG and DDB) independently marked quotations containing pieces of data that were relevant for analysis. These quotations were compared, and a consensus document that contained all relevant pieces of data for each transcript was created. Each quotation was then classified as containing information about negative opinions, positive opinions, facilitating factors, barriers, or solutions. After this first rough classification into categories, the quotations were further interpreted by a single researcher who identified specific codes (eg, “people are naturally inclined to respond to rewards”). These specific codes were then again reviewed by an independent second coder who agreed or disagreed with the identified codes. Through discussion, all disagreements were eventually solved, resulting in a list of 33 codes. These specific codes were first categorized into broader categories and finally assigned to one of four overarching themes that emerged (eg, “positive attitude toward rewards”). This process involved sorting and categorizing similar codes and retracting each step multiple times until each of the 33 codes was assigned to one of the four overarching themes. Although data were analyzed and themes were identified using thematic analysis, we additionally used a technique taken from content analysis and counted how often a piece of code was encountered. This helped us quantify how often a specific code was mentioned. For publication purposes, quotation examples were translated into English by 2 researchers (JVDG and DDB).

## Results

The following themes emerged: (1) positive attitude toward rewards, (2) too much focus on extrinsic aspects, (3) structure and form of the reward, and (4) characteristics of the target group.

### Positive Attitude Toward Rewards

The first important finding is that HCPs generally show high acceptance of and hold positive attitudes toward a financial incentive system. Although one respondent explicitly rejected the idea of rewarding people and 2 others were doubtful whether it would be a good idea, the majority of respondents (13/16, 81%) expressed positive attitudes. Often mentioned was that a healthy lifestyle is challenging for patients with CVD and HCPs believe that a reward might help to provide a necessary nudge (7/16, 44%). HCPs believe that external commitment from a reward system would be effective in supporting sustained healthy living (7/16, 44%). One respondent explained this by emphasizing the affirmation that the delivery of a reward might provide:

I certainly think that receiving a reward provides patients with a sort of feedback. That feedback gives them the recognition that they are doing something right. I certainly believe that it has potential.

HCP1

Furthermore, respondents believed rewards to be effective because people are naturally inclined to respond to rewards (4/16, 25%): “A reward system always works because that is how people are wired” [HCP2].

### Too Much Focus on Extrinsic Aspects

The second important finding is that half of the HCPs (8/16, 50%) believe that a financial reward system might put too much emphasis on extrinsic motivational aspects of lifestyle change. This could draw attention away from the intrinsically rewarding aspects of healthy living, such as feeling more fit (3/16, 19%). A proposed solution would be to always emphasize the intrinsic aspects and health benefits in addition to providing extrinsic rewards (2/16, 13%). Furthermore, providers of a reward system risk paternalizing patients by communicating to them what they should be doing and thus threatening patients’ autonomy (2/16, 13%): “I would have objections when a reward system moves in the direction of conditioning people to act like circus animals” [HCP3]. According to the respondents, this focus on rewards could then also lead patients to overstrain themselves or even manipulate to get rewards (3/16, 19%). A possible solution would be to support autonomy by allowing freedom of choice in rewards (3/16, 19%). In addition, a sentiment strongly felt by some was that patients should find motivation from within, instead of needing a reward system (3/16, 19%):

Patients should know better, especially patients that get acute hospitalizations [...] they should investigate their own behavior and change that

HCP4

Finally, almost half of the HCPs expected the motivating effect of rewards to fade out over time (7/16, 44%).

### Structure and Form of the Reward

The structure and form of the reward itself were deemed relevant for the success of implementing a reward system by 6 respondents. Related to the form of the reward, HCPs were worried that when rewards were not large enough, they would not have the intended motivating effect (2/16, 13%). Although one respondent argued for allowing as much freedom of choice as possible (eg, by letting participants choose how to be rewarded; 1/16, 6%), another emphasized that to avoid supporting unhealthy behavior, only rewards should be provided that are in line with the behavior needed to attain them (eg, running for a discount on running shoes; 1/16, 6%). Another concern was that the rules for how rewards could be earned would be made too complex or nontransparent (2/16, 13%). Finally, two respondents suggested to stimulate engagement with a reward system by using social interaction (2/16, 13%):

I do think that a reward system would work best when you make it social [...] You are not going to celebrate when nobody is watching. While if there are many people watching—suppose I won a trophy at the Australian Open and 20.000 people are watching—I am going to scream from the top of my lungs to celebrate!

HCP1

### Characteristics of the Target Group

The respondents mentioned several concerns regarding the characteristics of subgroups of the population with CVD that could interfere with the successful implementation of a reward system. As patients with CVD are generally older, respondents are worried that some will have trouble using the technology necessary to measure their lifestyle behavior and receive the rewards (4/16, 25%): “A problem will be a lack of digital know-how, so logging into the system will be an issue, especially for the elderly” [HCP5]. This issue might be diminished by targeting a younger, more digitally literate population (2/16, 13%). Another issue is that respondents see 2 subgroups of patients who might not benefit from receiving rewards: (1) the already highly motivated individuals who will not receive additional motivation from being offered a reward system (1/16, 6%) and (2) the not-at-all motivated who will not respond to anything that is offered (1/16, 6%). Finally, respondents argued that a reward system risks rewarding the already successful (who do not need extra motivation) while punishing (and thus demotivating) nonachievers (4/16, 25%):

[...] how do you deal with situations where people do not achieve their goal? This could of course have multiple different reasons and in that situation, people are in fact being punished.

HCP6

### Key Concerns and Related Solutions

Whenever concerns were mentioned, we also asked for possible solutions. Therefore, in Textbox 1, we summarize the main concerns and related solutions suggested by the HCPs during the interviews.

**Key concerns and suggested solutions for implementing a financial incentive system.**

**Concerns**

- Focusing too much on extrinsic rewards
- Disengagement when rewards are insignificant, nontailored, or longitudinally provided
- Paternalization and threatening autonomy
- Lack of digital literacy in target group

**Suggested Solutions**

- Emphasize intrinsic aspects of healthy living while giving extrinsic rewards
- Integrate social aspects to increase engagement with rewards
- Support autonomy by allowing freedom of choice in rewards
- Focus on a target group that can work with the necessary technology

## Discussion

### Principal Findings

This is the first study to investigate the acceptability of a financial incentive system among Dutch CVD HCPs. Furthermore, we explored the barriers and facilitators of its implementation. The HCPs in our sample generally showed high acceptance of a reward system for healthy living for patients with CVD. This finding is consistent with the existing literature that also showed, among HCPs in the United States, high acceptance of a reward system for healthy living in CVD management [10]. The level of acceptability we found is also in line with the idea that attitudes are not necessarily negative but depend on contextual factors such as how the incentive is designed and whom it targets [9,12,17]. With regard to these contextual factors, Giles et al [9] and Promberger et al [17] found that acceptability was higher when incentives were perceived as more effective (“pay them if it works”). In line with these findings, this study indeed found that many respondents perceived financial incentives as an effective intervention, which might have been related to the relatively high acceptance that was found. Furthermore, Giles et al [11] showed that policy makers perceive financial incentives as more acceptable when they target vulnerable subgroups. People with CVD might be considered a vulnerable group, which might explain why it is more acceptable to reward them for healthy living [11]. Similarly, the high acceptability we found could be explained by previous research, suggesting that voucher-based incentives—as presented to HCPs in this research—are more acceptable than cash incentives [12,18]. Finally, previous research has shown that privately funded incentives are considered more acceptable than publicly funded incentives [12,13]. The way the reward system for this study was explained to participants might have implied private funding, and thus high acceptance, because we mentioned the use of reward points that could be exchanged for discounts at commercial product and service suppliers.

Notwithstanding the generally positive evaluations we found, several important concerns emerged within the themes that were discussed. HCPs were concerned that rewards could lead to focusing too much on the extrinsic aspects of lifestyle change and could threaten autonomy. This might have negative effects, such as increasing pressure on patients with CVD and possibly leading to manipulation for rewards. These concerns can be interpreted as a reflection of ethical objections among HCPs in our sample. This finding is in line with the ethical reflection by Ashcroft, which states that financial incentives can be perceived as paternalistic, coercive, involve bribery, or undermine the agency of the person [7]. At a more practical level, concerns emerged around disengagement with rewards in the long term. For those looking to implement a financial incentive system that aims to be in place long term, it is important to take these practical concerns into account. For example, as mentioned by the respondents, through integrating social aspects in the incentive system.

An important new finding that emerged from this work is that digital literacy in the target population might be an obstacle to implementing a reward system for healthy living in CVD management. The use of digital technology is necessary to objectively measure goal progress and provide associated rewards. As the onset of CVD generally occurs at an older age, patients with CVD are expected to have lower digital literacy. For those looking to implement a financial incentive system that targets a less digitally literate group (eg, patients with CVD) and aims to be in place long term, it appears important to take these practical concerns into account. This obstacle could be overcome by either focusing on a subsection of younger, more technologically savvy participants or by simplifying the technological solutions to accommodate a larger group of patients with CVD. Future research should investigate whether patients with CVD recognize this obstacle and what they see as viable solutions. Developing a reward system in cocreation with patients with CVD can help simplify the technological solution and match its complexity to the digital literacy of the intended users. On the basis of the answers of the HCPs in this study, and in line with what other authors found [12], we propose that both ethical and practical concerns should be mitigated through thoughtful incentive design in cocreation with patients with CVD.

A limitation of this study is the possibility that HCPs opinions on using a financial incentive system were influenced by preceding discussions (as described in the *Method*s section) about other themes related to lifestyle change. More specifically, having thought about obstacles in providing support for lifestyle changes in patients with CVD might have primed HCPs to the necessity of accepting alternative intervention supplements such as financial incentives. This could have led to an overestimation of acceptability to levels that would not be found when the financial incentive system would have been discussed in isolation. In addition, because we used convenience sampling and contacted individuals and organizations that were associated with the BENEFIT consortium, opinions on a reward system could be more positive than would otherwise be the case. Although the high acceptability that we found is consistent with existing research, some caution in drawing firm conclusions with regard to acceptability is warranted. Another consideration is that before asking HCPs about their opinions, we provided a concrete example of what a financial incentive system might look like. Therefore, the findings of this study should be interpreted in relation to a voucher-based financial incentive system (points to be exchanged for goods and services), and generalizing these insights to other forms of financial incentive systems should be done with caution. Finally, the sample used in this study was heterogeneous and relatively small. Integrating the perspectives of HCPs from various disciplines and institutes across the Netherlands ensures a broad view of opinions but makes in-depth discussions about discipline-specific or institute-specific insights impossible. Future research that aims to support local implementation could use a more homogenous sample and a fine-grained approach to overcome this.

### Conclusions

This study mapped the opinions of Dutch HCPs working in CVD care. In line with existing studies on different populations outside the Netherlands, Dutch HCPs in general showed high acceptance of a financial incentive system for healthy living for patients with CVD. However, there are important concerns that should be considered when designing a financial incentive system. In particular, the concern about digital literacy in the target group is a novel finding and warrants further investigation. According to the HCPs interviewed in this study, suggested solutions to overcome concerns around a financial incentive system for patients with CVD are (1) emphasizing intrinsic aspects of healthy living while giving extrinsic rewards, (2) integrating social aspects to increase engagement with rewards, (3) supporting autonomy by allowing freedom of choice in rewards, and (4) aiming for a target group that can work with the necessary technology.

The high level of acceptability we found among Dutch HCPs provides support for further investigation and development of a reward system for CVD, as will be pursued in the BENEFIT consortium. Finally, although investigating HCPs’ opinions is an important first step, it is also important to know the opinions of the patients that would be targeted by financial incentives. Therefore, in the next step, we will validate the current insights among Dutch patients with CVD. The aim of these cocreation efforts is to contribute to the design of financial incentive interventions to better fit the needs of both clinicians and patients in CVD care.

## Appendix

Multimedia Appendix 1 Overview of the interview guide.

## Abbreviations

CVD: cardiovascular disease
HCP: health care professional
NWO: Nederlandse Organisatie voor Wetenschappelijk Onderzoek
