# Supplementary material for: Financial Incentives for Healthy Living for Patients With Cardiac Disease From the Perspective of Health Care Professionals: Interview Study
Source: JMIR Cardio. 2021 Aug 30;5(2):e27867. doi: 10.2196/27867 (PMC8438607; doi:10.2196/27867)
Supplement: Multimedia Appendix 1 [file cardio_v5i2e27867_app1.docx]

| Multimedia appendix 1. Overview of the interview guide | |
| --- | --- |
| Interview theme | Questions |
| *Facilitating and impeding factors in the uptake of and adherence to a healthy lifestyle for CVD patients* | *What do CVD patients need to do in their home environment to achieve sustainable lifestyle change?*  *What things that seem to work well for CVD patients in changing their lifestyle?*  *What impedes CVD patients in changing their lifestyle?*  *What solutions do CVD patients have for these barriers?* |
| *Facilitating and impeding factors in providing lifestyle support to CVD patients* | *How do you provide lifestyle support to CVD patients?*  *What works well in providing lifestyle support?*  *What impedes providing lifestyle support?*  *What solutions do you have for these barriers?* |
| *Stakeholders that are involved in providing lifestyle support to CVD patients* | *What do you, as a healthcare professional, need to better provide lifestyle support to CVD patients?*  *With whom do you cooperate in providing lifestyle support to CVD patients?* |
| *Facilitating and impeding factors in using eHealth to provide lifestyle support to CVD patients* | *What things go well in your use of eHealth to provide lifestyle support to CVD patients?*  *What impedes your use of eHealth to provide lifestyle support to CVD patients?*  *What solutions do you have for these barriers?*  *What do you, as a healthcare professional, need to better make use of eHealth to provide lifestyle support to CVD patients?* |
| *Facilitating and impeding factors in using wearables and sensors to provide lifestyle support to CVD patients* | *To what extent do you use wearables and sensors to provide lifestyle support to CVD patients?*  *What things go well in your use of wearables and sensors to provide lifestyle support to CVD patients?*  *What impedes your use of wearables and sensors to provide lifestyle support to CVD patients?*  *What solutions do you have for these barriers?* |
| Facilitating and impeding factors in using a financial incentive system to support lifestyle change in CVD patients | **What is your opinion on using an incentive system?**  **What could be obstacles for using an incentive system?**  **What could be solutions to facilitate the use of an incentive system?** |
